# Supplementary material for: Age-related differences in psychopathology within sex chromosome trisomies
Source: medRxiv. 2024 Nov 23:2024.11.22.24317803. Preprint. [Version 1] doi: 10.1101/2024.11.22.24317803 (PMC11601772; doi:10.1101/2024.11.22.24317803)
Supplement: Supplement 1 [file media-1.pdf]

	Total	XX	XY	XXX	XXY	XYY	Group Comparisons		
							Comparing all groups	SCTs vs. controls	Comparing SCT groups only
**Federal Race Category: N(%)**									
Asian	11 (2.2)	3 (2.0)	3 (2.0)	3 (6.0)	1 (1.1)	1 (1.7)	$\chi2$= 35.87*	$\chi2$= 34.99**	$\chi2$= 9.94
Black	34 (6.7)	17 (11.3)	13 (8.5)	0 (0)	4 (4.3)	0 (0)			
American Indian / Alaska Native	1 (0.2)	0 (0)	0 (0)	0 (0)	1 (1.1)	0 (0)			
White	424 (84)	123 (81.5)	120 (78.4)	44 (88.0)	81 (88.0)	56 (95.0)			
More than one race	33 (6.5)	6 (4.0)	17 (11.1)	3 (6.0)	5 (5.4)	2 (3.4)			
Unknown / not reported	2 (0.4)	2 (1.3)	0 (0)	0 (0)	0 (0)	0 (0)			
**Federal Ethnicity Category: N(%)**									
Hispanic	34 (6.7)	10 (6.6)	6 (4)	4 (8)	9 (9.8)	5 (8.5)	$\chi2$= 9.23	$\chi2$= 3.60	$\chi2$= 2.58
Non-Hispanic	468 (92.7)	141 (93.4)	146 (95.4)	46 (92)	81 (88)	54 (91.5)			
Unknown	3 (0.6)	0 (0)	1 (0.7)	0 (0)	2 (2.2)				

Supplementary Table 1 Additional demographic variables * $p < .05$, ** $p < .001$

CBCL Scale	CBCL T-score by group, M(SD)				
	XX	XY	XXX	XXY	XYY
anxdep	51 (0.5)	51.3 (0.5)	60.3 (1.1)*	57.9 (0.8)*	59.7 (1.0)*
withdep	51.5 (0.5)	52.0 (0.5)	61.7 (1.1)*	58.9 (0.8)*	61.3 (1.0)*
somatic	52.2 (0.5)	52.3 (0.5)	62.6 (1.1)*	59.2 (0.9)*	63.3 (1.0)*
social	50.6 (0.5)	51.2 (0.5)	63.5 (1.0)*	60.7 (0.8)*	68.1 (0.9)*
thought	51.1 (0.5)	51.7 (0.5)	60.5 (1.0)*	60.0 (0.8)*	66.0 (1.0)*
attention	50.9(0.5)	51.4 (0.5)	67.1 (1.1)*	60.9 (0.8)*	68.6 (1.0)*
rulebreak	51.3 (0.4)	51.4 (0.4)	56.6 (0.9)*	56.1 (0.7)*	59.0 (0.8)*
aggressive	50.7 (0.5)	51.0 (0.5)	58.1 (1.0)*	56.5 (0.8)*	62.6 (0.9)*
internal	42.5 (0.8)	43.6 (0.8)	61.7 (1.6)*	57.6 (1.3)*	62.2 (1.5)*
external	42.0 (0.8)	42.6 (0.8)	53.9 (1.6)*	53.8 (1.2)*	59.4 (1.5)*
totalprob	40.0 (0.8)	41.3 (0.7)	61.4 (1.6)*	58.7 (1.2)*	65.8 (1.4)*

Supplementary Table 2 Mean CBCL T-score for each CBCL dimension in each karyotype group (estimated at mean age of the combined cohort). Asterisk denotes Tukey post hoc adjusted *p*-value for comparison of each SCT group's CBCL T-score distribution relative to their respective gonadal control group ($p < .05$).

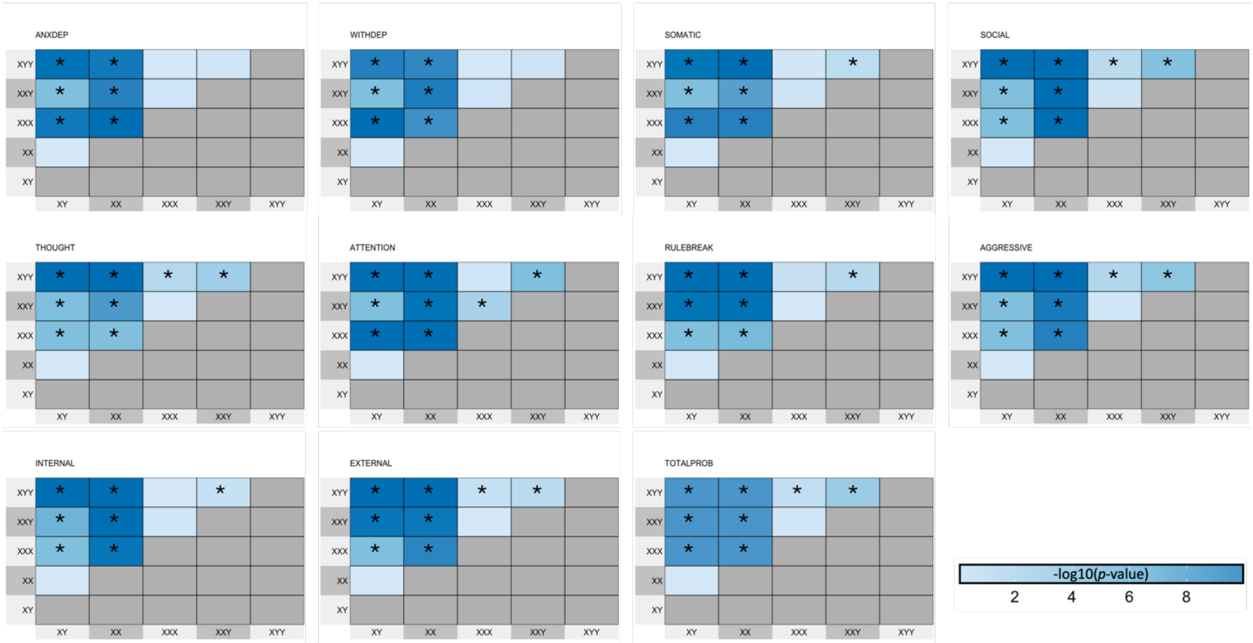

Supplementary Fig. 1 Pairwise tests for inter-group differences in mean CBCL T-score for each CBCL dimension. Colors encode the -log10(*p*-value) from Tukey test for each group difference. Asterisks denote statistically significant contrasts (adjusted *p*-value < .05). Directions of group differences are detailed in Supplementary Table 2.
